# Barriers and enabling structural forces affecting access to antiretroviral therapy in Nigeria

**DOI:** 10.1186/s12889-023-17271-6

**Published:** 2024-01-06

**Authors:** Kingsley Oturu, Oonagh O’Brien, Philomena I. Ozo-Eson

**Affiliations:** 1 University of Strathclyde, Glasgow, Scotland UK; 2 Institute for Global Health and Development, Queen Margaret University Edinburgh, Edinburgh, UK; 3 Departmment of Sociology, University of Abuja, Abuja, Nigeria

**Keywords:** Structural forces, Antiretroviral therapy, Access, HIV/AIDS, Nigeria

## Abstract

**Background:**

Access to antiretroviral therapy (ART) helps to improve quality of life and reduces the spread of HIV. However, while a lot of studies focus on supply factors, such as resources for the purchase of antiretroviral drugs, demand and structural forces are not given much emphasis. In this paper it is argued that structural forces shape the way people access antiretroviral therapy in Nigeria.

**Methods:**

A Grounded Theory methodology was undertaken in the research. Semi structured qualitative interviews were administered to select people living with HIV/AIDS in Nigeria. This was facilitated by the Network of People Living with HIV/AIDS in Nigeria (NEPWHAN) to understand their perspectives with regard to barriers and enablers to ART access in Nigeria. Thirty persons living with HIV/AIDS were interviewed and recorded. The interview recordings were transcribed and coded using a constructionist epistemological approach. This was triangulated with results of preliminary and secondary literature review analysis.

**Results:**

In this research, the participants discussed structural forces (barriers and enablers) that influenced how they accessed ART. These included economic factors such as poverty that enabled transactional sex. Unequal gender relations and perceptions influenced how they accessed ART. The participants’ belief in ‘God’ and religious activities such as ‘prayer’ and the use of ‘traditional medicine’ had an impact on how and when they accessed ART. Political activity at the international, national, and local levels influenced access to ART as well as resources. The individual’s familial, social, and organisational connections also influenced their ease of accessing ART.

**Conclusions:**

This study identifies structural forces that affect access to antiretroviral therapy and provides recommendations on how they can be harnessed to enable improved access to ART and consequently improved health.

## Background

In the literature on HIV, increasing attention and importance have been given to structural forces and the ways they influence HIV prevention. Structural forces such as gender inequality and poverty are very important factors that drive the HIV epidemic [1]. These structural forces can act as barriers to individually oriented HIV prevention and care services and the adoption of HIV-preventive behaviours [2]. For example, fear of HIV/AIDS-related stigma and discrimination discourages people from seeking HIV counselling and testing.

The aim of this paper is to explore the experiences of People Living with HIV/AIDS (PLWHA) accessing ART programmes in Nigeria. In this paper, the role that barriers and enabling structural forces play in influencing ART access are also explored. For the purpose of this paper, structural forces are social, cultural, organisational, economic, legal or policy features in societies that influence health behaviour. They are structural because they are intangible and have an influence on human behaviour. Research undertaken using data from Africa, Asia and South America, suggests that stigma, discrimination, poverty and unequal gender roles stand as major barriers to accessing ART programmes in resource poor settings [3].

This paper focuses on four structural forces which were found to be most relevant in this current study. These structural forces are the economy, politics, gender and religion/spirituality. This paper is divided into 3 sections. In the first section, structural forces are introduced. In the second section, structural forces are related to access to ART. Structural forces do not only play important roles in HIV transmission, but they can also play vital roles in the way people access HIV treatment services. In the last section, conclusions are made. In this paper, it is argued that access processes do not occur in a contextual vacuum. They occur within structural or contextual environments which shape how and when people access HIV treatment. A change in one aspect of the structural spectrum can affect other areas.

Furthermore, structural forces that influence access to HIV treatment are context specific. What is an important structural factor in one country may be irrelevant or have lower significance in another. For example, in the United Kingdom, ART programmes and laboratory services are provided free of charge to the user as they are funded by the UK National Health Service (NHS). In contrast, the Nigerian health system uses a pay for service mechanism. It may be easier to access HIV treatment in contexts where all services are free compared to contexts where some fees need to be paid. However, organisational arrangements may also affect the ease of access.

Even though donor funded ART programmes in Nigeria are free, due to the fact that they are often limited to government hospitals, there are usually large numbers of people attempting to access treatment in small numbers of public hospitals located in urban areas. This leads to long waiting times. Most of the discussions on structural forces in HIV focus on HIV prevention as structural forces have an important role to play in the rate of new cases of HIV infection compared to individual psychological strategies [4].

Research in Rio de Janeiro, Brazil suggests that policies that promote a positive socio-political environment combined with the social mobilisation of citizens contribute to improved access to HIV treatment [5]. Similarly, structural labelling of HIV positive persons as not being trust worthy or unproductive within the health care system environment tends to reduce access to treatment [6]. Combining the structural intervention of providing housing for homeless HIV positive persons with treatment initiatives, assists in increasing access to ART programmes in the USA [7]. Similarly, in Zimbabwe different structural barriers have a stronger impact in reducing access to ART programmes than individualistic factors [8].

Structural forces have a dualistic role. They may assist HIV positive people’s access to treatment or may stand as barriers. For example, political support has been hailed as one of the reasons why Uganda made significant progress in reducing its HIV prevalence. Conversely, the high prevalence rate of HIV in South Africa has been associated with a lack of political support [9].

Of all the structural forces, economic forces appear to be the most important. For the purpose of this paper, economic factors are a conglomerate of resources that an individual or group can command to access HIV treatment. Nigeria has the second largest economy in sub-Saharan Africa, generating 41% of the region’s GDP [10]. However, the large GDP does not reflect the inequality that exists between the poor and the rich. Economic inequality within countries is one of the drivers of the HIV pandemic [11]. ‘Structural violence’ has been coined as a term to explain how poor people are structurally prone to disease and detrimental life outcomes [12]. The poor are at an increased risk of contracting HIV and other infectious diseases. They may be at increased risk due to lack of access to health information, rural urban migration and consequent transactional sex and peer pressure to adopt risky sexual lifestyles.

Among other factors, poverty may also influence health seeking behaviour and access to ART. It will require strong health care systems and innovative multi-disciplinary approaches, which take cognisance of the local context. These programmes need strong structural theoretical underpinnings to guide the strategies for them to be effective.

Four out of ten in the Nigerian population live below the poverty line (less than a dollar a day). Nigeria is experiencing sluggish economic growth and low human capital that exacerbates poverty [13]. The result of dependency and the demise of manufacturing capacity of developing countries is a poor economic outlook. Lack of access to factors of production such as land, capital and human resources also contributes to poverty. At the global level, contributing to national poverty is the unfavourable terms of trade in the global market. Globalisation predicated on market led growth runs the risk of increasing social exclusion as poor people are not supported by advantaged groups [14]. These issues could also occur at country level as poor countries are not supported adequately to develop.

The inability to provide ART to HIV positive persons may also affect the economy negatively as they are too ill to engage in economically productive activity [15]. With a poor macroeconomic outlook, there is a corresponding reduced capacity of individual citizens to afford ART at the microeconomic level [11]. The person with little or no education finds it difficult to get a sufficiently high paying job and remains in poverty. The person who is HIV positive and is unable to afford treatment loses income and contributes to the reduction of the nation’s economic productivity. The presence of untreated sexually transmitted diseases also contributes to the spread of HIV [16].

Politics is a structural issue that can influence how resources are allocated for health programmes. Without their role, ART may not have been as accessible as they are today in Nigeria. With low pharmaceutical manufacturing ability, Nigeria is dependent on ART drug supply from pharmaceutical companies based in other countries. The ART drugs are often patented and their production and exportation are governed by the Trade-Related Intellectual Property Rights (TRIPS) agreement of the World Trade Organisation [16].

The TRIPS may provide a structural barrier to affordable ART as TRIPS allow pharmaceutical companies that produce ART to monopolize producing new ART for about 20 years. This contributes to an increase in the cost of patented drugs, and therefore limits their access to developing countries [17]. The international donor agencies wield a lot of influence in the provision of ART [18]. The TRIPS are negotiated by members of the World Trade Organisation. These negotiations tend to favour developed countries where the ideas tend to emanate from [19]. The intellectual property processes may assist in limiting access to ART as poor countries are dependent on richer countries for the production of the ART [16].

The funding does not come without strings attached as there are conditionalities. With PEPFAR funds for example, the drugs initially had to be procured from pharmaceutical companies based in the United States of America with their higher prices rather than cheaper generics. There were also ‘moral’ conditions that were applied.

These included advocating for abstinence from extra marital sex and the preclusion of PEPFAR funds being used for or to promote abortions [16].

Two developments within the World Trade Organization (WTO) have played a role in potentially improving access to generic ART. These are the 2001 ‘Doha Declaration on TRIPs and Public Health’ and the ‘30 August Decision’ of the WTO General Council in 2003. They allow flexibilities within the TRIPS agreement that allows low income countries to invoke compulsory licences or certificates of notification for producing the ART locally for public health reasons. However, these agreements have had a limited effect, largely because, not many developing countries are not making use of the loopholes or flexibilities (such as patent pooling) as they either do not have any ART pharmaceutical industry or do not have a sufficiently large manufacturing capacity to satisfy the local demand for ART [19].

In some cases, International Donor Agencies (IDAs) act as global social connectors by mediating between the pharmaceutical companies that produce drugs and the developing countries that need drugs. In other words, they provide the funding for the rolling out of ART. Global social connectors include philanthropic foundations (such as the Bill and Melinda Gates Foundation), international non-governmental organisations and IDAs. These social connectors have been instrumental in negotiating with pharmaceutical companies to donate free ART to African countries [16]. These policies are occasionally put on the national agenda following pressure from global actors (social connectors) [17]. A modified general framework that examines the strategic positioning of different donor agencies and provides an overview of how donor agencies are important in influencing structural change in developing countries is depicted in Table 1 [20].

**Table 1:** Adapted donor strategic framework for International Donor Agencies [20]

	**World Health Organisation** **(WHO)**	**United Kingdom Department for International Development** **(DFID)**	**United States Agency for International Development** **(USAID/PEPFAR)**
Development Perspective	Structuralist	Structuralist	Neo-liberal
Focus	Social groups/communities		Private sector, non-governmental organizations
Goal	Equalised outcomes	Pro-poor, Pro-vulnerable groups	Freedom, choice
Means of distribution	Egalitarian	Redistribution	Free market
State involvement	Maximalist	Partialist	Minimalist
Health care providers	Works through state structures	Initially worked mainly with civil society, but policies now favour working with state structures	NGOs, civil society, private hospitals and the state
Access to health & social care	Right of citizenship, not dependent on individual income or wealth	Requires contribution from the community	Ability to pay
Sustainability	Meets needs of present without compromising the ability of future generation to meet their own needs	Meets the need of the present and provides foundation for future generation to meet their own needs	Fast, of high quality. Meets the need of the present, but may not be sustainable when funds are not forthcoming

There are also studies that have shown a link between agenda setting by politicians in power and levels of allocation of resources for health or other priorities such as malaria [21]. It is also important to tackle HIV as not only a medical issue but also as a social issue. The National Agency for the Control of AIDS in Nigeria is funded mainly by the GFATM [22, 23].

Gender inequality in access to wealth also makes women more prone to being poor and at a greater risk of acquiring HIV than men. Gender inequality means women are less able to command resources and assets compared to men in order to safeguard their health. Furthermore, gender issues affect ART access programmes as some women depend on men for financial resources and permission in order to access treatment. [24].

For the purpose of this research, spirituality is broadly defined as belief in prayer, medication, having faith in God and drawing strength on those beliefs. For the purpose of this paper, religion is the belief in a Supreme Being (God), supernatural powers or forces that have an influence on human destiny. In Nigeria, the cultural perception of illnesses as having evil spiritual undertones (such as witchcraft) was strongly associated with the use of spiritual healing churches as an alternative to modern health care in Western Nigeria [25, 26]. This implies that these religious perceptions need to be taken into consideration when rolling out ART programmes. Part of the problems in tackling HIV/AIDS in Africa is the tendency of the Western biomedical scientific world to dismiss or ridicule these beliefs in preference for the general hegemony of scientific knowledge that links HIV with AIDS [27].

In order to have effective ART access programmes, it is important to take cognisance of the important influence of religion. The important role of religion in health has been well documented as it may positively influence health in three ways [28]. Firstly, religious communities encourage health enhancing behaviours (such as having monogamous sexual relationships). Secondly, religious meditation has been linked to psychological health benefits. Thirdly, the social dimension of religion and the social connections that emanate from religious practices may contribute to improved health. A lot of other confounding factors may also contribute to improved health.

In terms of psychological support of PLWHA, religion has also been found to be important. Research in the United States of America, suggests that greater involvement in spiritual activities has been associated with lower emotional distress in PLWHA [29]. Spirituality and religion may also help PLWHA to adjust and cope better psychologically [30]. Structural forces do play an important role in influencing how people access ART programmes [31].

## Methods: study setting and design

Abuja (the capital territory of Nigeria) was chosen as the project site for the following reasons: Firstly, it is a multicultural/multi-ethnic territory. Secondly, it had medium HIV prevalence rate. Thirdly, HIV support groups from where participants were recruited are present. Fourthly, Abuja has health facilities where HIV positive persons can access ART programmes. The research involved 2 qualitative phases. In the first phase, the literature on access to ART programme was reviewed. The second phase of research data collection in Nigeria was conducted through the use of semi structured interviews. This was through the Network of People Living with HIV and AIDS in Nigeria (NEPWHAN), that assisted PLWHA. The Network of People Living with HIV and AIDS in Nigeria (NEPWHAN) is a Non-Governmental Organization (NGO) that serves as a collective voice of PLWHA in Nigeria. The NGO empowers, strengthens and coordinates all support groups, state networks, constituencies, associations and organizations of PLWHA in Nigeria.

The research instrument used was an interview guide. The interview guide was piloted on the first 6 participants to check for appropriateness, cultural sensitivity and to see if they helped answer the research question. As explained in more detail in the analysis section of this paper, the pilot participants were included in the study, the number took cognizance of numbers usually needed to achieve theoretical saturation in similar Grounded Theory studies (which is 30). It is challenging anticipating all possible scenarios that could be tested. It is advocated that the pilot size should be at least 10% of parent/project study size [32].

The research approach had to be adapted iteratively to allow more open questions and follow the lead of the participants. The same interview guide was used throughout the research. The results of the literature review and the interviews were triangulated to provide a picture on how participants access treatment. Grounded theory was chosen as the preferred qualitative methodology as it is rigorous, iterative and useful in theoretical development.

## Participants’ characteristics

The target population are PLWHA who have experience of trying to access ART from the health care system. Inclusion criteria are: participants should have a positive HIV Elisa test, they should not be less than 18 years of age, they should have attempted successfully or unsuccessfully to access ART from the health facilities, they need to be physically fit to partake in the study, they should also be psychologically able to communicate and they should be Nigerians. Attempts were made as much as possible to ensure that there was equal representation of male and female participants. However, there are more female members in NEPWHAN and this is reflected in the male to female ratio of participants recruited for the study. The names used in this current paper are not names of participants. They are pseudonyms.

Participants for semi-structured interviews were recruited through the executives of the association of PLWHA (gatekeepers who they are familiar with), using leaflets (placed at the NEPWHAN office and telephone calls. Using the snowball technique, participants recruited through the leaflets and telephone calls were then involved in recruiting other participants for the study. This strategy also took cognisance of confidentiality, the sensitive nature of HIV and the observation that Africans tend to participate in studies when someone that they know introduces them to the study [33–35]. Attempts were made to overcome selection bias by asking recruiters not to only recruit people who are close to them or only people belonging to the association of PLWHA (NEPWHAN).

The characteristics (age, ethnicity, religion, gender, level of education and rural/urban residency) of the participants were also monitored throughout the research process. 30 Participants were recruited from the membership of a national association of people living with HIV/AIDS (PLWHA) in Nigeria. The sample size is determined by theoretical saturation. Theoretical saturation is the point whereby the addition of new data or information from the participants of the study does not bring any new contributions to the development of the emerging theory. This occurs when new participants are repeating information that already had been obtained from past participants. This usually occurs with a sample of 20 to 30 participants [36–38]. Theoretical sampling was employed. In theoretical sampling, participants are recruited purposively initially through telephone and advertisements on information leaflets. Subsequent participants were recruited who were able to give more information on categories of themes identified until no new themes emerged from the interviews [37, 38].

**Fig. 1:**
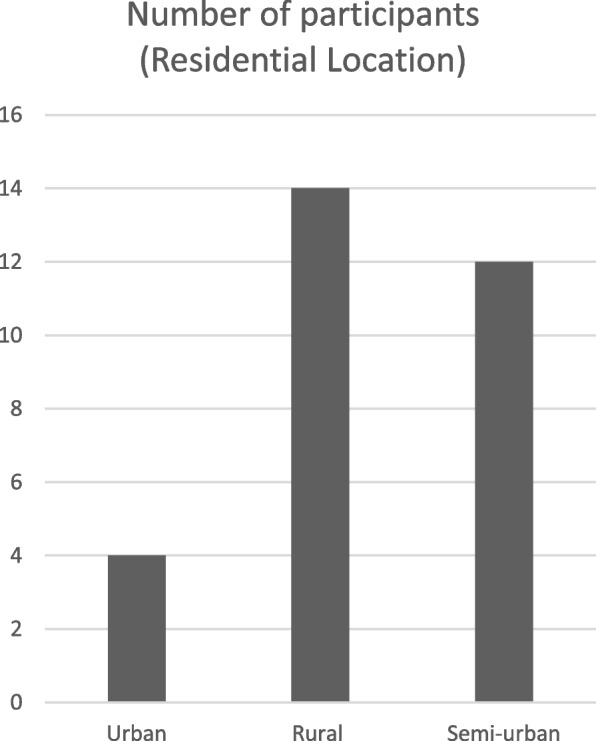
Graphic representation of the residential location of participants

**Fig. 2:**
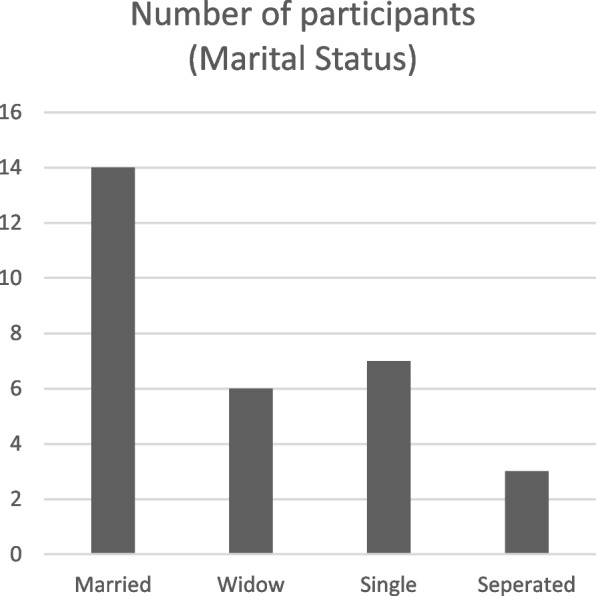
Graphic representation of the marital status of participants

**Fig. 3:**
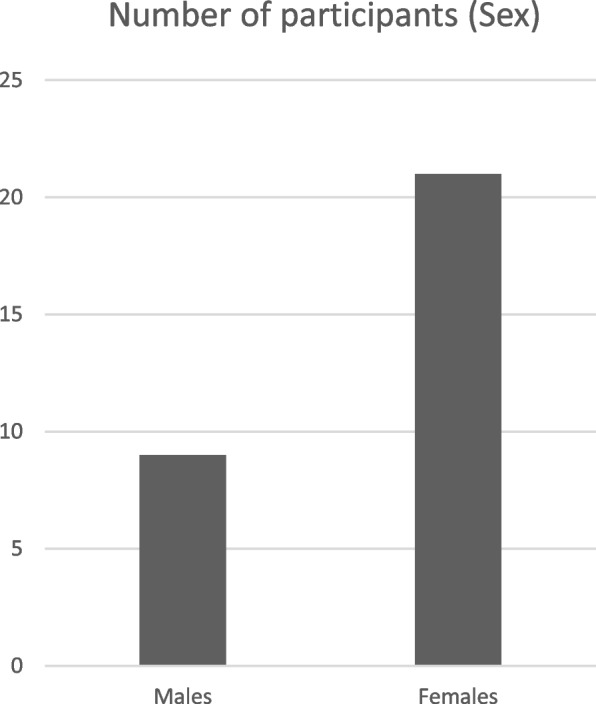
Graphic representation of the sex of research participants

Although most of the participants worked in the urban areas of Abuja, 14 were resident in rural areas, 12 in semi-urban areas and only 4 lived in urban areas. Most of the participants (46%) lived in rural areas while 41% of the participants lived in semi-urban areas (Fig. 1). They usually travelled from these areas to the ART centres to access their medication. Out of the 30 participants, 6 were single, 14 were married, 7 were widowed, while 3 were separated from their marital partners because of HIV infection (Fig. 2). In this research, attempts were made to ensure that both males and females were recruited. This is reflected in the sample recruited for the study. In total, 21 women and 9 men were recruited for the study (Fig. 3). Part of the reasons why more women were recruited, may hinge on the fact that more women belonged to HIV support groups than men.

## Data collection

The semi-structured interview was utilised as the primary method of data collection as it is easier to organise follow up interviews if issues are raised which need clarification. Interviews allow respondents to reconstruct events that they have passed through in their own words. Interviews allow access to a wider variety of people [39]. Questions were modified as interviews progressed, adjusting to the emphasis of issues that emerged from the research [40].

Research participants were informed that times and locations could be tailored to fit their convenience. However, all the interviews with participants took place at the conference room of the association of PLWHA in Abuja. Efforts were made to build up a rapport with the participants. This took socio-economic status, age, sex and culture into consideration. This included asking questions about how they were feeling and how they came to the interview site.

Following the introductions, the voluntary nature of the research was emphasized. The participants were given information sheets about the research. Participants were allowed to ask questions. Permission to record interviews and written consent were sought before proceeding with the interview [41]. As a sign of their consent, the participants voluntarily put their initials and signatures on the consent form. They were then assigned pseudonyms to protect their anonymity.

Interviews were limited to a maximum of one hour in order to prevent participant distress. The interview room was well ventilated. The participants had access to water or refreshments if needed. The structure of the interview was adapted as necessary if the participants needed to take a break, or postpone all or portions of the interview. They were given the option of having someone close to them available after the interview to offer support [42]. The in-depth interviews were digitally recorded and transcribed for comparative analysis. The process of ‘transcoding’ was developed in this research. During transcoding, the process of transcribing and coding occurs concurrently. Full transcriptions of all the recordings were performed. Each transcript took initially about 6–10 h to analyse with open coding. As the transcription process progressed, the analysis became faster lasting about 3–5 h. The initial transcripts were read fully in order to gain familiarisation with the data. Later transcripts were coded as concepts emerged during the transcribing process.

## Analysis

Detailed Grounded Theory analysis of the data was undertaken through open, selective and theoretical coding. For the purpose of this paper, coding is the process of classifying chunks of data with labels that describe and explain social processes [43, 44].

With open (initial) coding, themes derived from the analysis were labelled and categorized. Each category was delineated in terms of properties and dimensions [43]. Different texts were labelled with relevant codes as depicted by the data. Different incidents within the transcripts were compared with each other. Incidents in later transcripts were also compared with incidents in earlier ones iteratively. Using these comparisons, the properties and dimensions of categories were defined. Using comparative analysis, data were compared with data to find similarities and differences. Constant comparative analysis is undertaken by comparing data from earlier transcripts with those from later transcripts.

With selective coding a core category was identified and systematically related to other categories. The core category is the central phenomenon of interest that links all the other categories [45]. Relationships between categories were refined and developed. Categories were then integrated together. Codes that could be merged to explain higher level concepts are then organised into categories. As codes were selected, a code was identified that is central to the analysis and links all the various codes together. This is called the ‘core category’.

With theoretical coding, the identified core (stigma) was systematically related to other categories. Relationships between categories were refined and developed. Categories are then integrated together. A Grounded Theory is arrived at which is derived from the data. However, the steps involved in the Grounded Theory approach do not constitute a linear process. The process is iterative with a continuous interplay of data collection and analysis using the constant comparative method [46].

Analysis was aided with the use of Nvivo software which allows easy coding and analysis of data. It can save time in multiple coding and excel performs complex searches quickly and efficiently. It also allows easy transportation and sharing of files and the ability to create models linked to themes that they represent.

To help ensure the quality of the research analysis, a variety of procedures were employed. Firstly, data collection and analysis were done concurrently so that interpretation of the data was compared with experiences of participants who were later recruited. Secondly, developed concepts were cross checked with transcripts to ensure that they are grounded in the data. Thirdly, initial readings of transcripts were conducted independently and interpretations compared by different researchers. The first author was involved in data collection, analysis and coding. The second and third authors were independently involved in cross checking the research for appropriateness and thoroughness in data collection, analysis, and coding. Fourthly, member checking was undertaken to assure validity. Last but not least, findings were also triangulated with results of the literature review [36].

## Results

### Structural forces and HIV

Findings from this research indicate that with increased donor funding, access to ART has become easier. Results of this study also show that structural policies such as to the location of ART centres influence access to ART.

## Economic forces

The impact of structural forces on female sexual behaviour is highlighted by the response of Linda, a 27-year-old HIV positive female participant. She mentioned that if she is in desperate need of money, she will readily have unprotected sex with a stranger to get money for food and other amenities.

*‘‘Someone like me, I’m looking young. If I’m walking on the streets, you will not know that I’m HIV positive. If they call me….. He says he loves me. I will go. Because I know no how, he is going to give me something (i.e. money). Because at that spot, I needed that money and he calls me, I will give him. Even if he does not use condoms. You understand…’’ (Linda, 27-year-old female counsellor).*

As Mairi reports, incidental costs can serve as barriers to accessing ART programmes.

*‘‘Yes… They will have to travel to somewhere else to go and access drugs and that too also is a barrier to their consumption of drugs. Some of them will feel that they don’t have transportation…money to transport themselves to the place and some will feel the place is far and they will not be able to go.’’ (Mairi, 30-year-old hair dresser).*

Another participant had to stop his therapy temporarily when he ran out of money to pay for the ART.

*‘‘So, with my condition, the whole…the little money that I had on was exhausted and the period I was taken care up to a point because it was as if it was boiling…’’ (Jimmy, 37-year-old unemployed graduate).*

One of the participants (Sabrilla) was also able to buy some ART at a reduced price when the hospital ran out of stock from HIV positive people who ‘collect and sell’.

*‘‘I will see some people. Maybe they have collected and they are selling. So, I will buy for like 4000…4,000, 5000… Yes, they will want to sell, to use the money to buy food. Ehen… So, if I buy, if I go back…’’ (Sabrilla, 60-year-old nurse).*

Structural interventions within the health care system that may assist in improving access include the provision of financial assistance to HIV positive persons to cover their expenses. In Bayelsa State Nigeria, one of the participants reported increased uptake of ART when the governor of the state announced that HIV positive persons who access their ART will be provided with financial support to cover nutrition and travel expenses. The participants report that people who had to travel far to access treatment were often able to stay over at a relative’s house (saving accommodation costs). However, it is not sustainable for continuous access when the patient does not want to disclose that he/she is taking ART.

## Political forces

Part of the complex milieu of factors contributing to Nigeria’s economic problems is lack of the political leadership and good governance. For example, some of the participants interviewed reported that they had started ART but had to stop for months because they could not afford to pay for drug treatment. However, following pressure from international donor agencies (IDAs) and the provision of funding, drugs and delivery mechanisms, some of the participants later restarted.

Findings from this current research indicate that PEPFAR and the Global Fund to Fight AIDS, Tuberculosis and Malaria (GFATM) are the major donor agencies for ART programmes in Nigeria. Apart from donor agencies, global alliances of International non-governmental organisations and activists have been instrumental in putting access to ART on the policy agenda and in lobbying governments and pharmaceutical companies to make the drugs more accessible. Local activists were also involved in Nigeria lobbying and advocating for free ART for PLWHA.

*‘‘Initially, there was difficulty. The difficulty was because we had to pay. We had to pay. Even when the government own came before PEPFAR we were placed on a waiting list. So many of us…We were expecting then. We had to keep going to the hospital to find out if we had been enlisted.’’ (John, 48-year-old military officer).*

As one of the participants reported, local political decisions determine direction of flow for funds. With dependency on donor aid and loans for ART programme access, there is a feeling among the participants that the Nigerian government will not be able or willing to take up funding of treatment if donor agencies stop helping. As participants in this study indicates, it may be important for the Nigerian government to develop long term funding strategies for HIV activities, so that if external donor funding stops, the country can be able to cope.

The participants of the study were vocal about having an anti-discrimination bill that prevents PLWHA from being discriminated against. There were reports from the participants about people being refused jobs in banks or scholarships because of their HIV positive status.

As argued above, poor governance and poverty appear to play a role in impeding access to ART programmes. Economic factors are vital for access to ART programmes. However, there are other factors that affect access either directly or through effects on the economy. Stigma, politics, gender, and other social factors are intertwined in a complex milieu and influence access in wide ranging ways.

### Gendered forces and access to ART

Findings from this current research indicate that with the provision of free ART by the Nigerian government, women are more likely to access treatment than men.

*‘‘You see, when you go to hospitals, you see more of the women…But the women do (access their ART). The women do because even if they are pregnant and they go to the clinic, they must be tested’’ (Austin, 56-year-old engineer).*

More women in this study had access to ART through antenatal care (ANC) than through Prevention of Mother to Child Transmission of HIV (PMCT) programmes. As evidenced in the following quotes from interviewees, these programmes are quite effective in improving access to ART:

*‘‘No. Men don’t like going for treatment oh! Most of them don’t even want to go for test. The only thing that is there is the women. And what made the women to go for the test is maybe if they are pregnant and they went for ANC…’’ (Shekira, 19-year-old student).*

Participants in this study reported that men’s status is linked with their ability to cater for the financial wellbeing of their families (even if it means sacrificing their health). In contrast, women cared more about their health as evidenced by the following quote from Linda, a 27-year-old female counsellor:

*‘‘Because women. Ah! Women they too like life (giggles)… We like living life. You know... So, we go for treatment. Yeah’’*

Last but not least, female participants in this study mentioned that they feared what would happen to their children if they died of AIDS because they refused to access treatment. Consequently, most of the participants in support groups and/or who access ART openly are women. This social connection of women in support groups and women support groups has a multiplier effect in increasing self-esteem and bolstering women to access treatment.

However, there are some women who refuse to access treatment because of the risk associated with meeting someone they know at the ART centre and the resultant stigma as demonstrated by one of the female participants:

*‘‘…because of that fear, you know being afraid of stigmatisation… because I didn’t want anybody and moreover, my profession (to know) …So, you know and you know the way they put our clinic…. Immediately you step their people will know’’ (Sabrilla, 60-year-old nurse).*

Based on these motivating factors listed in the previous section, women in this study accessed HIV treatment more openly than men. In contrast, men tended to be afraid of what would happen if they met a friend or someone they knew as they were accessing treatment (organisational stigma).

*‘‘You see, the men…not all the men are ready to go out to access their drugs…Not all… They don’t want somebody to know or somebody to see that they have gone to STI clinic.’’ (Austin, 56-year-old engineer).*

As one of the interviewees in this study reports, some men feel that they can beat the infection with their own power and don’t need to take medication.

*‘‘Because some men, they think what is in their mind say they have power…they have ability to suppress…the sickness cannot affect them. They always boast…this thing can not affect them.’’ (Saulle, 43-year-old male driver).*

*‘‘They give preference to women because they say they are the weaker sex. So, whatever it is, they will always consider women first.’’ (Beatrice, 44-year-old construction worker).*

Most of the HIV positive female participants reported that their husbands were reluctant to go for an HIV test after they informed them of their HIV status. If a woman is taking her ART and her husband refuses to accept ART, the woman runs the risk of re infection and ART resistance. There were some reports from this study of men who negotiate with their wives to engage in ‘drug sharing’ in which the woman goes to the ART centre to collect the medication and then shares the drugs with her husband (increasing risk of ART resistance).

As indicated earlier, gender-based violence is also linked with a higher incidence of HIV infection. One of the participants was beaten to the point of unconsciousness by her husband. She recounts how many women receive violence because of their HIV status as in the quote below:

*‘‘But we are all positive women. Mostly divorced or thrown out of the house. We don’t have women that are with their husbands there. Mostly if you go there you will hear experience… Mostly they are women that are battered. They are thrown out of their home or their husbands died.’’ (Monica, 37-year-old social worker).*

*‘’When I left my husband, I did not just leave because of the HIV, because at a point I was like a problem to him and every day beating, beating. The last time I left was because he beat me to coma. I was in coma. So, when I got up, I was looking at myself as a ghost. I said ‘Ah ah! So, it’s true that if you are dying you will know but you can’t just help yourself.’ Because I went blank. When I got up, I was like ‘Am I still alive?’ So that day I just decide, if I had died it would have been a story. Let me just stay alive. I discovered that he was tired and he didn’t really want to stay with a positive person. So, there’s no point forcing myself on him. ‘’ (Monica, 37- year old social worker).*

*‘‘There are some women that their husband driven them way. They pursue them…this support group, this month that passed. This month that we are*

*Entering…the first Saturday of the month, they went to Gbonsa. OK, as you go, a girl delivered a baby like this. The husband has driven, pursued her. That is how they sleep outside. See her body…kai! mosquitoes nearly kill her. Kai!’’ (Asha, 39-year-old petty trader).*

The findings of this research indicate that HIV positive women are at higher risk of gender-based violence. They are also deterred from seeking redress because of fear of stigma and the protracted judiciary system. Although the HIV diagnosis increases the risk of gender-based violence, this did not deter the women in this study from continuing to access HIV treatment.

HIV has the potential of destroying family structures. Some couples separated or divorced ‘peacefully’ following disclosure of the positive HIV status. One of the three females (Monica) was HIV positive while her husband was HIV negative. However, two of the research participants (1 male and 1 female) remained with their HIV negative partners despite being ‘sero-discordant’ couples. Seven of the participants (6 females and 1 male) lost their spouses who died of HIV infection.

Some men experienced loss of esteem in accessing ART programmes through job loss as a result of long absence from work as they access their treatment. This may be further exacerbated by the societal belief that strongly religious people should not be sick. Due to the religious nature of African societies, it will be important that men be involved in planning, implementing, and supporting ART access programmes.

## Religious forces

All the participants in the study were religious and believed that their faith in God helped them to access treatment. This is evidenced in the following quote from one of the interviewees:

*‘‘God helps them to access treatment. That’s the mentality of Nigerians. 80% of Nigerians believe that everything is from God.’’ (Tama, 45-year-old manager)*

*‘‘Yes. Before I cried very well. The thing worried me. I refused to tell anybody. I spent sleepless night for 2 days. My heart will pain me. I said ‘Jesus, I need your peace.’ I was so desperate. I did not know who I will tell such a terrible news to. So, I just told Jesus that I need your peace. It was like my heart will cut. Yes.’’ (Martha, 35-year-old teacher).*

*‘‘And God is unquestionable. He heals who He chooses to heal. The Bible says that He has mercy on who He wants to have mercy. So, all we can pray and believe it and claim what God has said to you in the Bible. It’s well… I pray… I fast (Monica, 37-year-old female civil servant).*

Some were already religious before becoming HIV positive while others became more religious in response to their diagnosis as demonstrated in the following quotes:

*‘’Halima: I have to come close to God and beg God. I have to be going to church constantly I now…I gave my life to Christ…I’m not supposed to be known…with a man before getting married. I’m supposed to be…abstain before getting married….’ (Halima, 37-year-old unemployed female).*

The thought that ‘God is in control’ helped them cope in their experience. The national president of one of the support groups in Nigeria highlights the point that Nigerians use their faith as a coping mechanism.

*‘‘People’s belief in God plays a major role in helping them cope with their HIV diagnosis and access treatment. Nigeria is a very religious country. When there is no more hope, people turn to God. I believe that I am being kept by divine healing of God as I am not on ART but am still healthy’’ (Tama, 45-year-old manager).*

In Nigeria, there is a strong belief in the spiritual aetiology of HIV infection that is often not recognised by health care practitioners. For example, one of the participants indicated that she believed she was HIV positive because she refused to marry her former boyfriend who placed a curse on her for disappointing him. Refusing to recognise patients’ spiritual beliefs could alienate patients from the health care worker.

Findings from this current study indicate that the stress that emanates from the ‘fear of dying of HIV’ can be very great and perceived as adding to the burden of poor health. Belief in God as well as psychological and social support are all coping mechanisms that participants in this study utilised in overcoming their fear. This belief is supported by a resource person employed in this study who reports that although her HIV positive sister was on ART, it was the fear of dying that killed her and not the ART.

*‘’It’s the fear. The fear killed her. Even though she was on ART and had her family to support her, she had a strong fear of dying of HIV. It is the fear that killed her.’’ (Glade, 36-year-old teacher).*

Another resource person for this current study mentioned that his taking ‘immune boosting herbs and his religious disposition may play a role in helping him overcome HIV. Similarly, some participants who indicated that being spiritual or religious helped them overcome different barriers to access ART. One of the participants reported that at the time when access to ART was very difficult in Nigeria, it was his faith in God that helped him overcome the different barriers to access treatment.

*‘‘So, it was difficult… but I was believing God that if I come, He will make a way and I will be on the list. Fortunately for me, getting to national hospital, they start the procedure.’’ (Jimmy, 37-year-old unemployed graduate).*

Findings from research participants of this current research, indicate that religion may serve as a platform to promote the welfare of vulnerable people living with HIV/AIDS as demonstrated by activities of religious organisations (such as the Catholic Action Committee on AIDS and the Redeemed Christian Church of God) who provide financial and nutritional support to PLWHA.

*‘‘Yes, we believe that God is real and God is existing but the only thing that is there, put your faith in action. Put it in practice but don’t say because God is alive or God is there for you, then you know what to do and you are not doing it. God cannot come down from heaven and solve your problems. He will send one or two persons.’’ (Shekira, 19-year-old student).*

## Discussion

This study investigates the structural forces that influence access to ART in Nigeria. The results show that most of the Nigerian government ART centres are in the urban areas, making it difficult for some people to access treatment. The findings are consistent with those in South Africa and British Columbia where people living in rural areas found it difficult travelling to urban areas to access ART [47, 48]. It is difficult to quantify the significance of each of these structural forces. One factor may affect another in complex ways that are dependent on time, geography, and politics. For example, political decisions at the global level may affect positive foreign cash flow to Nigeria for aid which may improve access to ART in Nigeria. Conversely, a change in political regime at the country level to military rule may be met by negative reactions at the global level (such as stopping financial aid and/or supply of free ART).

As demonstrated in the results section, the provision of free ART made it easier for PLWHA to access HIV treatment. It is important that political pressure be continued to ensure that ART access is on the political agenda. ART access programmes might not have been successful without the added pressure from global social connectors (such as international donor agencies (IDAs)). The findings are consistent with those in qualitative research which suggests that global financial policies have an influence on ART access [49]. The participants in this current study were aware that their ART programme access was possible due to the funds provided by IDAs. Although the donor agencies have moved in to help provide ART, it is vital that they have a long-term strategy. The IDAs also need the cooperation and technical assistance of local political structures for their strategies to be effective. It is important that strategic partnerships continue with local NGOs and religious organisations to enable access to ART. The religious leaders wield a lot of influence on influencing HIV related stigma [50]. They could help people access treatment by supporting them in prayers and linking them to where they can get the ART. On the other hand, they can stand as a barrier to access by telling members of their congregation to rely on prayers instead of accessing the ART.

Structural forces such as poverty could have a far more profound effect on the health behaviour of people than the impact of receiving health care information. As shown in the results section, poverty had the power to drive one of the participants (Linda) to undertake risky sexual behaviour, even though she was aware of HIV and the sexual mode of transmission. Findings from this current study indicate that to access treatment, the patient considers, microeconomic considerations such as the cost of accommodation, loss of income from absence from work, transportation costs, consultation costs and the social (stigma) cost of being seen accessing treatment from the ART centre. The patient considers the opportunity costs of taking time out of other competing commitments to access the ART.

As suggested in the results section with illustrations from Bayelsa state, the provision of financial aid to PLWHA to support travel expenses helped improve ART access in that part of Nigeria. The findings are consistent with research in Uganda, which suggest that transportation costs can impede access to ART [51]. The findings of this research are also consistent with research in Tanzania where integration of food support in ART programmes helped improve access to ART [52]. Structural interventions that could assist in improving access could include provision of financial support for persons living with HIV/AIDS.

The findings of this current research demonstrate that women are more likely to access ART for several reasons. Firstly, women are offered the choice of doing an HIV test during antenatal care (ANC). This contrasts with most of the male participants who only went for an HIV test as a last resort when they were ill and other forms of alternative therapy had failed. Secondly, a lot of donor agencies support Prevention of Mother to Child Transmission of HIV (PMCT) programmes and women are placed on ART if found to be HIV positive. Thirdly, women are more likely to engage with good health seeking behaviour such as early ART initiation compared to men. This consistent with research undertaken in Ethiopia [53]. However, planning programmes for women without taking gender relations between men and women into consideration could be counterproductive. It is important to ensure men are also encouraged to access ART early as well to prevent the spread of the HIV/AIDS pandemic.

## Conclusion

From this current research it is concluded that structural forces play a major role in how and when PLWHA access ART programmes. It is important to consider structural forces when planning and implementing ART access programmes and also the structural mechanisms that will facilitate access to ART in resource poor settings.

With regard to *economic forces*, the provision of financial support for transportation and nutrition for those who cannot afford it may assist in enabling access to ART programmes. Developing social service units within health care systems that are sensitive to support people living with HIV/AIDS could be useful.

In terms of *political forces*, it is imperative to mount political pressure continually on governments so that HIV/AIDS continues to be placed on the agenda and governments should continue to fund and provide support for ART programmes. Legislation is also important to protect HIV positive persons from being discriminated against. Moreover, lessons learnt from tackling the AIDS pandemic may also be useful in dealing with other emerging infectious diseases.

With regard to *gender forces*, there is need to consider gender relations in the roll out of ART programmes. Gender strategies need to be tailored to fit the context in a way that will enable access to ART. Transforming gendered norms in Nigeria could be challenging and the media may serve as a useful tool in bringing about that transformation. From this research, it was discovered that more women tend to access ARV services than men. More women also belonged to HIV support groups than men. As suggested in this research, there is need for greater involvement of men in HIV programmes. More research is needed on barriers that make it difficult for men to access treatment.

Taking cognisance of *religious forces,* strategic partnerships and alliances with religious leaders/institutions is key as they can influence access to ART programmes. Religious organisations can also refer people to where they can access ART programmes as people will be more likely to access ART if encouraged by their religious leaders. Strategic partnerships between religious organisations and the health care system may potentially lead to better collaborative support for HIV positive persons.

Structural forces play a major role in preventing or spreading of HIV infections and are also vital in enabling or preventing access to ARV therapy. There are no easy answers with regard to managing structural forces and enabling access to ART programmes. It is vital to highlight the fact that structural forces are constantly changing with time and are influenced by globalisation.

Further research may be needed on the role of IDAs on access to ART, the power dynamics and how they can influence policy. The interaction of the different structural forces on access to ART may be context dependent. Further research that provides a greater understanding of the weight of each factor on access to ART and their levels of interdependency is also needed.

## Data Availability

The data that support the findings of this study are available on request from the corresponding author, [K.O.]. The data are not publicly available due to sensitive nature of HIV infection and their containing information that could compromise the privacy of research participants.
